# The distribution of groundwater uranium in Chintamani village, Karnataka, India

**DOI:** 10.12688/f1000research.162525.1

**Published:** 2025-03-24

**Authors:** Sadashiva Rampur, Mahesh Kumar V. K., Pavan R. Pelli, Senjuti Sarkar, Samayeta Pramanik, Upama Majumder, Shravanthi S., Bhavana Meenakshi T., Srinidhi G. Santhanakrishnan, Rushi Pendem, Tanushree Ghosh, Deepesh Nagarajan

**Affiliations:** 1 Department of Biotechnology, Faculty of Life and Allied Health Sciences, M.S. Ramaiah University of Applied Sciences, Bengaluru, Karnataka, 560054, India

**Keywords:** Uranium, heavy metals, hydrogeology, aquifer contamination

## Abstract

**Background:**

Chintamani village, Chikkaballapura district, Karnataka, India was found to possess high aquifer uranium concentrations. Ge- ologically, Chintamani village is located on bedrock that is rich in elements like potassium (K) that naturally contain high levels of radioactive elements, such as uranium and thorium, due to the presence of alkali-feldspar granites and gneisses. Aquifer de- pletion has caused the concentration of these elements in groundwater to increase over time, posing a potential health hazard to the residents of Chintamani village.

**Methods:**

Here, we report the sampling of groundwater from 12 borewells located in Chintamani village in between the period of August 2024 to December 2024. We observed groundwater uranium concentrations of 0.02 ppm to 8.64 ppm. Data for borewell depth, the quantity of total dissolved solids (TDS), and the elemental composition of TDS is also reported. We observed a statistically significant spatial distribution of uranium concentrations in Chintamani village. Borewells possessing the highest observed concentrations of uranium were clustered towards the northwestern region of the village.

**Conclusions:**

This dataset is expected to serve as a resource for guiding potential remediation efforts in these locations.

## Introduction

Uranium in groundwater primarily originates from natural geological sources, particularly from uranium-rich bedrock. Uranium may occur at high concentrations in intrusive igneous rocks, including two-mica granite, calc-alkaline granites, and alkalic plutonic rocks at concentrations of 3–300 ppm. ^1^ The crystal structures of igneous minerals like biotite, muscovite, K/Na-feldspar, and quartz may incorporate uranium in the percent range. ^2, 3^ Uranium is also found in sedimentary rocks such as shales (2-4 ppm), bauxite (11.4 ppm) and lignite ( *<*50-80 ppm). ^1^

Deep, water-stressed aquifers are frequently in contact with uranium-rich bedrock, enabling uranium to leach out into the surrounding groundwater. While this is a geogenic process, human activities can further exacerbate uranium contamination in groundwater. The use of nitrate-based fertilizers enhances the mobility of uranium by making it more soluble in water. ^4^ Additionally, over-extraction of groundwater lowers the water table, requiring deeper borewells to be drilled into uranium-rich bedrock. Diverse regions across the world experiencing water stress also display higher groundwater uranium content. Uranium concentrations in San Joaquin Valley, California, were observed to exceed federal and state drinking water standards of ≤ 30 ppb. ^5^ Groundwater in the Datong Basin, China, displayed uranium concentrations of *<*0.02–288 ppb, with a mean of 24 ppb. ^6^ Likewise, high groundwater uranium concentrations have been reported in southern Finland, Germany, Portugal, ^7^ Japan, Mongolia, Uzbekistan and, India. ^8^

The World Health Organization (WHO) recommends a maximum uranium concentration of 30 ppb in drinking water to minimize health risks. ^9^ Although uranium is weakly radioactive, its primary health risk stems from its chemical toxicity rather than its radioactivity. Chronic exposure to uranium-contaminated water is associated with nephrotoxicity, ^10, 11^ adverse effects on bone function and development, ^12^ reproductive and developmental toxicity. ^13^

In this data note, we report data obtained from 12 borewells in Chintamani village, Chikkaballapura District, Karnataka, India. We report the uranyl concentrations (ICP-MS), and TDS elemental compositions (SEM-EDS) of groundwater obtained from all the wells sampled. A previous survey reported high concentrations of groundwater uranium from 73 borewells spread across 13 districts of Eastern Karnataka, ^14^ reporting high uranium concentrations of *>*1 ppm in Chitradurga, Tumkur, Kolar, and Chikkaballapura districts of southeastern Karnataka. The bedrock in these districts is composed primarily of Neoarchean granites, gneisses, and migmaties. ^14^ The borewells we sampled displayed uranium concentrations ranging from 0.02 ppm to 8.64 ppm, confirming the concentration ranges reported in the previous survey. Furthermore, we report spatial localization of groundwater uranium. We observed significant differences in the distribution of uranium concentrations within the local water table. A significantly higher concentration of uranium was observed in borewells clustered at the northeastern region of Chintamani village, meriting further investigation of the village’s local geological features.

## Methods

### Sample collection

Groundwater samples from Chintamani village were collected using the purge and sample method. Each borewell was pumped for five minutes to remove stagnant water from the well casing and tubing. After purging, a 2 L water sample was collected in a clean polypropylene bottle.

### Total dissolved solids (TDS) quantification

For every borewell sample, 1 L of water was dried in a hot-air oven at 80 ^°^C in a clean Borosil ^®^ borosilicate 1L glass beaker until only the salt residue remained. This residue was weighed using a Shimadzu AXT224R precision balance (least count = 0.1 mg) and subjected to SEM-EDS in order to determine its elemental composition.

### Inductively coupled plasma mass spectrometry (ICP-MS)

ICP-MS experiments to quantify uranium concentration in the parts per million (ppm) range was performed by Eurofins Scientific India using a PerkinElmer ^®^ 350X instrument. A 20-40 mL sample from each borewell was submitted. The sample was acidified with nitric acid to adjust the pH to ≤2. The instrument was set to detect elemental uranium concentrations. Raw data were interpreted using the Syngistix™ software (version 4.0, PerkinElmer ^®^). Raw data may also be interpreted using openMS. ICP-MS reports for each sample can be found in Supplementary Dataset S1. ^15^

### Scanning electron microscopy - energy-dispersive X-ray spectroscopy (SEM-EDS)

An FEI (Field Electron and Ion Company) Quanta 200 scanning electron microscope at Icon Labs Pvt. Ltd., Mumbai was used to perform SEM-EDS experiments. Samples were observed under a low vacuum mode at 20 kV, and with a chamber pressure of 65 Pascal. SEM-EDS has a least count of 0.1% (by weight) and cannot detect elements below this concentration. SEM-EDS spectra and reports quantifying the elemental composition of each sample can be found in Supplementary Dataset S2. ^16^

### Data representation and statistical analyses

**Figure 1.**
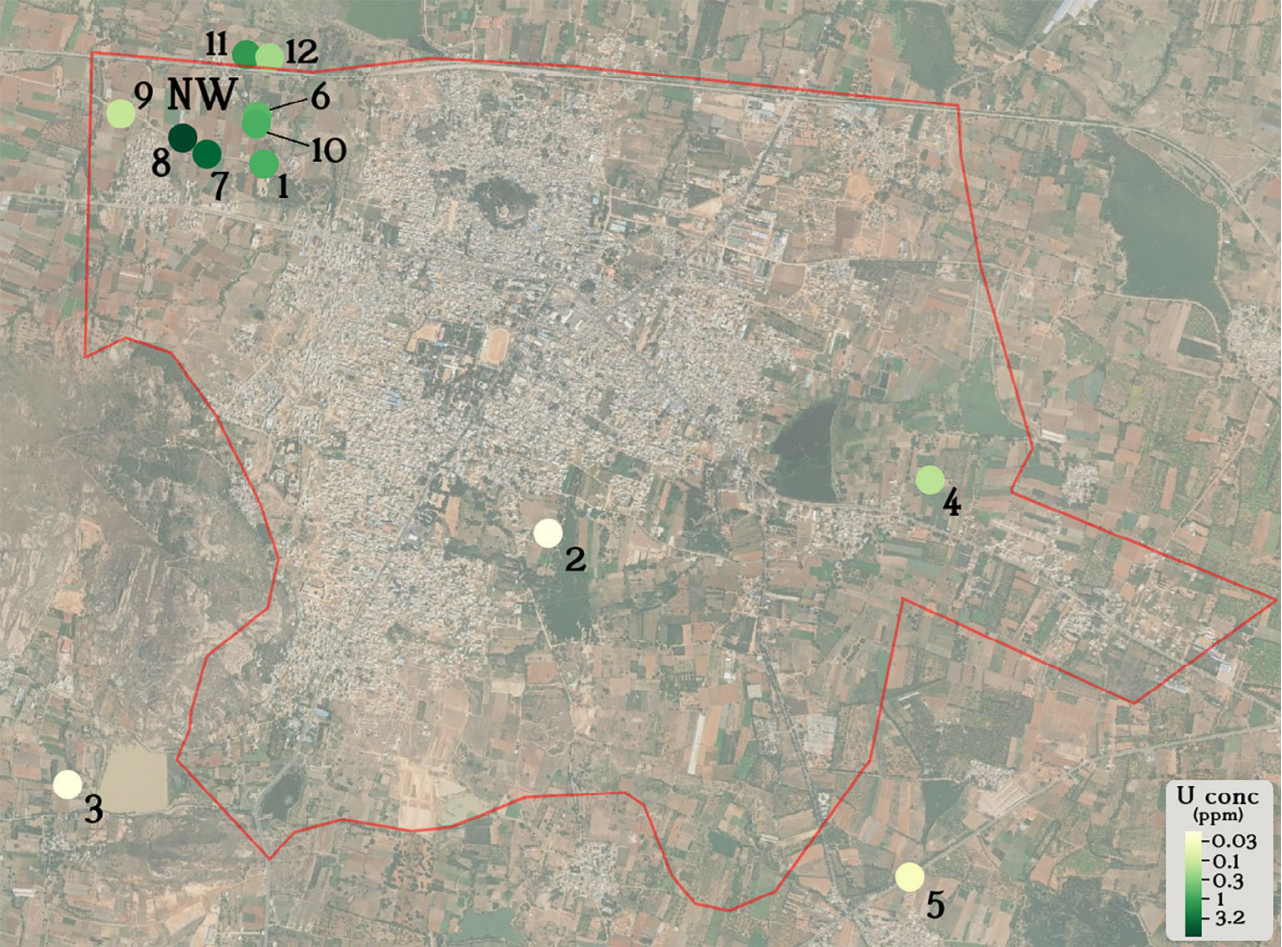
Groundwater uranium distribution in Chintamani village (red boundary).

All statistical analyses were performed using the R programming language (version 4.4.2). The Leaflet package ^17^ was used to generate a physical map of Chintamani village ( Figure 1). The Welch 2-sample T-test (one-tailed) was used to determine whether there existed a statistically significant difference between uranyl concentrations in different spatial locations in Chintamani village ( Figure 1). This was performed using the function t.test(). Pearson’s correlation coefficient and the statistical significance (p-value) between uranyl concentrations and the other variables discussed was calculated using the function cor.test() ( Tables 1, 2).

**Table 1.** Uranium concentrations (in ppm) for groundwater collected from Chintamani village.

Borewell name	Collection date	Latitude	Longitude	U (ppm)	Borewell depth (feet)	TDS (mg */*L)
Borewell 1	28Aug2024	13.407162	78.042081	0.771	1250	789.60
Borewell 2	28Aug2024	13.391223	78.054693	0.018	1550	192.30
Borewell 3	28Aug2024	13.380344	78.033369	0.02	1600	183.40
Borewell 4	28Aug2024	13.393505	78.071632	0.143	350	1009.60
Borewell 5	28Aug2024	13.376384	78.07072	0.035	No data	548.00
Borewell 6	30Nov2024	13.409183	78.04178	0.595	No data	2099.50
Borewell 7	21Dec2024	13.4075572	78.0395479	4.15	1200	542.70
Borewell 8	21Dec2024	13.4082796	78.038472	8.64	2000	706.20
Borewell 9	21Dec2024	13.4093118	78.0357153	0.12	200	686.40
Borewell 10	21Dec2024	13.4088807	78.0417362	0.74	1500	844.50
Borewell 11	21Dec2024	13.4118821	78.0413087	1.27	150	638.50
Borewell 12	21Dec2024	13.4117252	78.0423317	0.21	200	891.10
			**Mean**	1.39	1000.00	760.98
			**SD**	2.55	702.38	490.99
			**Corr. coeff.**		0.49	-0.05
			**P-value **		0.15	0.88

**Table 2.** Elemental composition of TDS obtained after drying groundwater samples collected from Chintamani village.

Borewell name	Data type	C	N	O	S	Na	Mg	Si	Cl	K	Ca	Al	Rh
Borewell 1	Mean (mg/L)	162.10	40.43	361.08	8.29	34.35	35.37	11.29	64.04	1.97	68.93	0.00	0.00
(0.771 ppm U)	SD (mg/L)	15.41	4.59	23.80	5.88	16.09	3.16	2.90	15.18	0.53	9.70	0.00	0.00
	Mean (%)	20.53	5.12	45.73	1.05	4.35	4.48	1.43	8.11	0.25	8.73	0.00	0.00
	SD (%)	1.95	0.58	3.01	0.74	2.04	0.40	0.37	1.92	0.07	1.23	0.00	0.00
Borewell 2	Mean (mg/L)	21.29	14.07	98.55	1.73	18.27	9.23	7.53	8.71	0.52	12.45	0.00	0.00
(0.018 ppm U)	SD (mg/L)	3.21	4.63	10.94	0.49	8.33	1.56	1.59	8.71	0.19	3.96	0.00	0.00
	Mean (%)	11.07	7.32	51.25	0.90	9.50	4.80	3.92	4.53	0.27	6.47	0.00	0.00
	SD (%)	1.67	2.41	5.69	0.26	4.33	0.81	0.82	4.53	0.10	2.06	0.00	0.00
Borewell 3	Mean (mg/L)	68.70	3.01	80.65	0.55	17.39	0.93	3.73	2.31	0.14	5.79	0.00	0.00
(0.02 ppm U)	SD (mg/L)	8.88	1.10	5.70	0.22	4.94	0.42	2.04	1.23	0.08	4.68	0.00	0.00
	Mean (%)	37.46	1.64	43.98	0.30	9.48	0.51	2.03	1.26	0.08	3.16	0.00	0.00
	SD (%)	4.84	0.60	3.11	0.12	2.70	0.23	1.11	0.67	0.05	2.55	0.00	0.00
Borewell 4	Mean (mg/L)	188.21	28.61	419.15	8.58	63.44	45.35	13.63	157.83	1.51	82.96	0.25	0.00
(0.143 ppm U)	SD (mg/L)	41.52	5.70	53.89	2.94	34.20	8.07	2.94	39.23	0.91	31.75	0.87	0.00
	Mean (%)	18.64	2.83	41.52	0.85	6.28	4.49	1.35	15.63	0.15	8.22	0.03	0.00
	SD (%)	4.11	0.56	5.34	0.29	3.39	0.80	0.29	3.89	0.09	3.14	0.09	0.00
Borewell 5	Mean (mg/L)	75.72	55.75	268.32	4.58	29.29	21.87	11.51	36.12	3.44	41.10	0.00	0.00
(0.035 ppm U)	SD (mg/L)	33.53	4.78	31.94	8.20	21.23	4.24	3.47	21.90	0.85	14.98	0.00	0.00
	Mean (%)	13.82	10.17	48.96	0.84	5.35	3.99	2.10	6.59	0.63	7.50	0.00	0.00
	SD (%)	6.12	0.87	5.83	1.50	3.87	0.77	0.63	4.00	0.16	2.73	0.00	0.00
Borewell 6	Mean (mg/L)	231.15	147.59	961.15	24.77	167.33	82.09	31.7	253.2	5.88	195.88	0.00	0.00
(0.595 ppm U)	SD (mg/L)	50.25	14.1	93.99	4.31	84.42	10.23	4.52	71.37	1.51	60	0.00	0.00
	Mean (%)	11.01	7.03	45.78	1.18	7.97	3.91	1.51	12.06	0.28	9.33	0.00	0.00
	SD (%)	2.39	0.67	4.48	0.21	4.02	0.49	0.22	3.4	0.07	2.86	0.00	0.00
Borewell 7	Mean (mg/L)	111.31	27.57	251.16	3.85	14.06	31.91	6.57	79.94	1.57	14.98	0.00	0.00
(4.15 ppm U)	SD (mg/L)	41.24	5.52	18.57	1.47	5.17	7.76	1.87	24.42	0.36	5.11	0.00	0.00
	Mean (%)	20.51	5.08	46.28	0.71	2.59	5.88	1.21	14.73	0.29	2.76	0.00	0.00
	SD (%)	7.6	1.02	3.42	0.27	0.95	1.43	0.34	4.5	0.07	0.94	0.00	0.00
Borewell 8	Mean (mg/L)	91.24	47.39	331.77	10.73	43.29	37	10.38	80.01	4.03	50.42	0.00	0.00
(8.64 ppm U)	SD (mg/L)	31.86	3.37	19.24	2.06	12.97	4.26	1.22	18.42	0.63	10.59	0.00	0.00
	Mean (%)	12.92	6.71	46.98	1.52	6.13	5.24	1.47	11.33	0.57	7.14	0.00	0.00
	SD (%)	4.51	0.48	2.72	0.29	1.84	0.6	0.17	2.61	0.09	1.5	0.00	0.00
Borewell 9	Mean (mg/L)	103.58	54.36	316.02	9.95	51.34	26.7	12.08	67.27	1.65	40.5	0.00	3.02
(0.12 ppm U)	SD (mg/L)	56.39	10	39.49	3.09	20.6	6	3.75	27.88	0.63	13.71	0.00	9.93
	Mean (%)	15.09	7.92	46.04	1.45	7.48	3.89	1.76	9.8	0.24	5.9	0.00	0.44
	SD (%)	8.22	1.46	5.75	0.45	3	0.87	0.55	4.06	0.09	2	0.00	1.45
Borewell 10	Mean (mg/L)	165.78	41.3	370.31	8.11	44.08	27.87	7.26	81.16	1.69	96.95	0.00	0.00
(0.74 ppm U)	SD (mg/L)	32.51	14.35	39.66	3.48	19.81	7.44	2.11	28.41	0.72	28.74	0.00	0.00
	Mean (%)	19.63	4.89	43.85	0.96	5.22	3.3	0.86	9.61	0.2	11.48	0.00	0.00
	SD (%)	3.85	1.7	4.7	0.41	2.35	0.88	0.25	3.36	0.09	3.4	0.00	0.00
Borewell 11	Mean (mg/L)	251.7	0	226.54	10.41	56.57	15.45	7.85	52.87	0.77	16.47	0.00	0.00
(1.27 ppm U)	SD (mg/L)	54.04	0	78.41	11.54	31.48	8	4.96	41.63	0.76	21.52	0.00	0.00
	Mean (%)	39.42	0	35.48	1.63	8.86	2.42	1.23	8.28	0.12	2.58	0.00	0.00
	SD (%)	8.46	0	12.28	1.81	4.93	1.25	0.78	6.52	0.12	3.37	0.00	0.00
Borewell 12	Mean (mg/L)	84.03	45.89	445.46	16.22	37.96	36.62	11.76	82.16	2.05	129.03	0.00	0.00
(0.21 ppm U)	SD (mg/L)	32.29	7.36	43.55	13.65	31.12	7.08	3.52	32.96	0.55	43.37	0.00	0.00
	Mean (%)	9.43	5.15	49.99	1.82	4.26	4.11	1.32	9.22	0.23	14.48	0.00	0.00
	SD (%)	3.62	0.83	4.89	1.53	3.49	0.79	0.4	3.7	0.06	4.87	0.00	0.00
**Conc. (mg */*L)**	Mean (mg/L)	129.57	42.16	344.18	8.98	48.11	30.87	11.27	80.47	2.10	62.96	0.02	0.25
	SD (mg/L)	70.02	38.13	224.92	6.60	40.69	20.53	7.04	67.60	1.62	56.45	0.07	0.87
	Correlation	-0.27	-0.10	-0.20	-0.03	-0.25	0.05	-0.31	0.02	0.21	-0.22	-0.15	-0.16
	P-value	0.39	0.75	0.53	0.92	0.42	0.87	0.32	0.95	0.5	0.48	0.63	0.62
**Relative %**	Mean (%)	19.13	5.32	45.49	1.10	6.46	3.92	1.68	9.27	0.28	7.31	0.00	0.04
	SD (%)	9.82	2.82	4.15	0.44	2.23	1.39	0.789	4.03	0.17	3.55	0.01	0.13
	Correlation	-0.1	0.06	0.03	0.22	-0.28	0.45	-0.24	0.36	0.49	-0.2	-0.15	-0.16
	P-value	0.75	0.85	0.93	0.49	0.37	0.14	0.44	0.24	0.1	0.54	0.63	0.62

## Chintamani groundwater datasets

Groundwater from 12 borewells in Chintamani village, Chikkaballapura District, Karnataka, India were sampled from August 2024 to December, 2024. Initially, we collected groundwater samples from borewells 1-5 that were evenly distributed around the geographical area of Chintamani village. Groundwater from borewell 1 displayed the highest uranium concentration from this cohort (0.771 ppm U), leading us to sample groundwater from more borewells around borewell 1 in the northwestern region of Chintamani village. We found a statistically significant difference (p = 0.048, Welch 2-sample T-test, one-tailed) between the uranium concentration of groundwater in the northwestern region (NW, borewells 1, 6-12) compared to groundwater in the rest of Chintamani village (borewells 2-5).

Table 1 depicts uranium concentrations quantified using ICP-MS from these 12 groundwater samples. Uranium con- centrations ranged from 0.02 ppm (Borewell 3) to 8.64 ppm (Borewell 8). There exists a weak correlation (r = 0.49, Pearson’s coefficient) between uranium concentration and well depth. However, the correlation is not statistically significant (p = 0.14). ICP-MS reports quantifying uranium content for each sample can be found in Supplementary Dataset S1. ^15^

Table 2 represents the elemental composition of the total dissolved solids (TDS) obtained after drying groundwater samples collected from Chintamani village. The elemental composition of dried TDS was determined using SEM-EDS. Elemental composition is expressed in absolute terms (mg of element per liter of groundwater, mg */*L) and in relative terms (% composition compared to all other elements present in dried TDS). Pearson’s correlation coefficients are provided for both expressions of elemental composition by comparing the values for every element with the corresponding uranium concentration (in ppm) (refer Table 1). It was observed that the % composition of K (r = 0.49, p = 0.1) and Mg (r = 0.45, p = 0.14) were weakly correlated with uranium concentration, although these correlations were not statistically significant. SEM-EDS spectra and reports quantifying the elemental composition of each sample can be found in Supplementary Dataset S2. ^16^

## Conclusion

We have presented a dataset containing uranyl concentrations from groundwater obtained from 12 borewells across Chintamani village, Chikkaballapura district, Karnataka, India. Uranyl concentrations ranged from 0.018 ppm (borewell 2) to 8.64 ppm (borewell 8). We have also provided a dataset of the elemental compositions of TDS obtained from these groundwater samples. These datasets could potentially be used as resources for guiding remediation efforts in this region.

## Ethics and consent

Ethical consent and approval were not required.

## Author contributions

Authors Sadashiva Rampur, Mahesh Kumar V.K., and Pavan R. Pelli surveyed and collected water samples from Chintamani village. Authors Senjuti Sarkar, Samayeta Pramanik, Upama Majumdar, Shravanthi S., and Bhavana Meenakshi T. processed groundwater samples to quantify TDS content, and processed samples for SEM-EDS and ICP-MS experiments. Authors Srinidhi G. Santhanakrishnan and Rushi Pendem analyzed and interpreted all data. Authors Senjuti Sarkar, Tanushree Ghosh, and Deepesh Nagarajan conceived the project and designed all experiments. All authors took part in drafting the manuscript and provided final approval before submission.

## Data Availability

Data are available under the terms of the Creative Commons Attribution 4.0 International license (CC-BY 4.0). All raw data have been made publicly available for use by the research community. Repository name: Dataset S1: ICP-MS data for groundwater uranium concentration in ppm. https://doi.org/10.6084/m9.figshare.28491125.v1. ^15^ The project contains the following underlying data:
•
**dataset-s1.pdf** ICP-MS reports (generated by Eurofins India, Bangalore) for the uranium concentrations of groundwater samples from borewells 1-12 (reported in ppm). Water samples were collected from Chintamani village, Chikkaballapura district, Karnataka, India, during the period of August 2024 to December 2024. Repository name: Dataset S2: SEM-EDS spectra of groundwater TDS, https://doi.org/10.6084/m9.figshare.28491146.v1. ^16^ The project contains the following underlying data:
•
**dataset-s2.pdf** SEM-EDS spectra and reports (generated by Icon Labs Pvt. Ltd., Mumbai) for the elemental composition of total dissolved solids (TDS) obtained after drying groundwater samples from borewells 1-12. Water samples were collected from Chintamani village, Chikkaballapura district, Karnataka, India, during the period of August 2024 to December 2024.
