# Supplementary material for: The distribution of groundwater uranium in Chintamani village, Karnataka, India
Source: F1000Res. 2025 Aug 21;14:321. Originally published 2025 Mar 24. [Version 3] doi: 10.12688/f1000research.162525.3 (PMC12409252; doi:10.12688/f1000research.162525.3)
Supplement: Peer review history

# Reviewer report: Reviewer response for version 3

**DOI:** 10.5256/f1000research.185995.r407690

**Published:** 2025-09-03

**Authors:** Nilanjal Misra

**Affiliations:** 1 Bhabha Atomic Research Centre, Trombay, India

Manuscript is acceptable in its current form.

Are sufficient details of methods and materials provided to allow replication by others?

Partly

Is the rationale for creating the dataset(s) clearly described?

Partly

Are the datasets clearly presented in a useable and accessible format?

Partly

Are the protocols appropriate and is the work technically sound?

Partly

Reviewer Expertise:

Development of environmentally sustainable technologies for heavy metal ion remediation in ground water

I confirm that I have read this submission and believe that I have an appropriate level of expertise to confirm that it is of an acceptable scientific standard.

# Reviewer report: Reviewer response for version 2

**DOI:** 10.5256/f1000research.182340.r399030

**Published:** 2025-08-12

**Authors:** Nilanjal Misra

**Affiliations:** 1 Bhabha Atomic Research Centre, Trombay, India

Although the work is well intended, the data set presented is relatively small and concentrated to a particular zone to draw any significant conclusions about the pattern of U concentration distribution in the region. The determination of elemental composition and its impact on the actual objective of the study is not well defined. Since the authors have mentoned that nitrate based fertilizers are responsible for solubilizing U from bed-rocks, a corelation between the N content of the samples and the U content can be discussed briefly. The authors can specifiy how many samples from each borewell have been analyzed and provide the mean U concentration for each borewell along with respective SD.

Are sufficient details of methods and materials provided to allow replication by others?

Partly

Is the rationale for creating the dataset(s) clearly described?

Partly

Are the datasets clearly presented in a useable and accessible format?

Partly

Are the protocols appropriate and is the work technically sound?

Partly

Reviewer Expertise:

Development of environmentally sustainable technologies for heavy metal ion remediation in ground water

I confirm that I have read this submission and believe that I have an appropriate level of expertise to confirm that it is of an acceptable scientific standard, however I have significant reservations, as outlined above.

# Reviewer report: Reviewer response for version 2

**DOI:** 10.5256/f1000research.182340.r383555

**Published:** 2025-05-29

**Authors:** Daniel O. Omokpariola

**Affiliations:** 1 Nnamdi Azikiwe University, Awka, Anambra, Nigeria

Dear Authors,

 Please find the attached work and review them for further modification

 1. Scope and Novelty

 a. The study is confirmatory and does not clearly articulate what new insights it offers beyond previous surveys.

 b. The authors should clarify:

 i. Why this specific location was chosen.

 ii. Whether the data updates or expands on previous findings.

 iii. How this dataset can inform remediation or policy decisions.

 2. Scientific Context

 a. The introduction should include:

 i. A clearer rationale for repeating uranium measurements in this region.

 ii. A brief overview of potential exposure pathways (e.g., drinking water, food chain).

 iii. A summary of known health risks from uranium exposure (e.g., nephrotoxicity, reproductive effects).

 3. Interpretation of Results

 a. The conclusion is too brief and lacks interpretation.

 b. The authors should describe the spatial distribution of uranium (e.g., “localized in the NW region”).

 c. Discuss whether the observed concentrations exceed WHO or Indian drinking water standards.

 d. Highlight any environmental or public health implications.

 4. Methodological Clarifications

 a.  The “Sample collection” section should include:

 i. GPS coordinates of all borewells (only partially included in Table 1).

 ii. Clarification on how borewells were selected (random, purposive, based on prior data?).

 iii. The use of “t.test()” in the text should be corrected or explained more clearly.

 5. Data Presentation

 a. The tables are comprehensive but dense.

 b. Consider summarizing key findings in a visual format (e.g., heatmap, scatter plot).

 c. Highlight borewells that exceed safety thresholds.

 d. A spatial map showing uranium concentration gradients would enhance clarity.

 6. Language and Style

 Minor grammatical and typographical issues should be corrected:

 Example: “Forevery” → “For every”

 Example: “hot-air ovenat” → “hot-air oven at”

 Example: “t.test()” → should be formatted correctly or described in plain language.

 Further Suggestions

 To enhance the manuscript’s scientific value and clarity:

 a. Expand the conclusion to include:

 b. A summary of spatial trends.

 c. Implications for public health or groundwater management.

 d. Recommendations for future research or monitoring.

 e. Add a brief section on limitations:

 f. Small sample size (12 borewells).

 g. Lack of temporal data (single sampling period).

 h. No direct health or exposure assessment.

 Include a visual summary (e.g., map or infographic) to improve accessibility.

Are sufficient details of methods and materials provided to allow replication by others?

Partly

Is the rationale for creating the dataset(s) clearly described?

Yes

Are the datasets clearly presented in a useable and accessible format?

Yes

Are the protocols appropriate and is the work technically sound?

Yes

Reviewer Expertise:

Environmental Chemistry and Toxicology; Remote Sensing; Atmospheric and Water chemistry; Risk assessment and Project Management

I confirm that I have read this submission and believe that I have an appropriate level of expertise to confirm that it is of an acceptable scientific standard, however I have significant reservations, as outlined above.

# Response

**Published:** 2025-08-01

**Authors:** Deepesh Nagarajan

Dear Authors,

 Please find the attached work and review them for further modification

AUTHOR RESPONSE: We thank the reviewer for his expert comments, and have addressed all the concerns raised below.

 1. Scope and Novelty

 a. The study is confirmatory and does not clearly articulate what new insights it offers beyond previous surveys.

 b. The authors should clarify:

 i. Why this specific location was chosen.

AUTHOR RESPONSE: The site selection was based on accessibility and its proximity to our university. We have now mentioned this in the Introduction section of the manuscript.

 ii. Whether the data updates or expands on previous findings.

AUTHOR RESPONSE: The data confirms and expands on previous findings due to our larger sample size (n=12), spatial groundwater uranium map withing Chintamai village (Figure 1), and the use of a more rigorous method (ICP-MS) to quantify groundwater concentrations.

 iii. How this dataset can inform remediation or policy decisions.

AUTHOR RESPONSE: The raw data along with our kernel density estimate (Figure 1) of groundwater uranium concentrations divides Chintamani village into groundwater zones possessing different groundwater uranium concentrations. Borewells and areas possessing higher groundwater uranium concentrations may be assigned a higher priority for remediation. This is now mentioned in the conclusion section.

 “ These datasets along with our kernel density estimate of groundwater uranium distributions (FIgure 1B) could potentially be used as resources for prioritizing guiding remediation efforts by displaying borewells and areas possessing the highest uranium concentrations. “

 2. Scientific Context

 a. The introduction should include:

 i. A clearer rationale for repeating uranium measurements in this region

 .

AUTHOR RESPONSE: We have elaborated on our rationale in the Introduction (paragraph 5).

 ii. A brief overview of potential exposure pathways (e.g., drinking water, food chain).

AUTHOR RESPONSE: We have outlined uranium exposure routes in the Introduction (paragraph 3 and 4).

 Introduction paragraph 3: Uranium adversely affects crops grown on soil irrigated with contaminated groundwater. Uranium’s phytotoxic effects include inhibition of photosynthesis, inhibition of plant growth, protein and lipid membrane oxidation, overproduction of reactive oxygen species, and DNA breakage [9]. Uranium primarily accumulates in root systems, with negligible amounts found in aerial parts of plants [10] and is therefore a greater concern for root and tuber crops.

 Introduction paragraph 4: Dermal exposure results in minimal toxicity, particularly if Uranium is in an insoluble form [11]. Uranium in groundwater is typically present as sparingly soluble uranyl carbonate complexes [12], minimizing the risk of absorption through the dermal route. Instead, oral consumption of uranium through drinking untreated uranium-contaminated groundwater is of more concern.

 iii. A summary of known health risks from uranium exposure (e.g., nephrotoxicity, reproductive effects).

AUTHOR RESPONSE: We have outlined the health risks associated with uranium exposure in the Introduction (paragraph 5).

 Introduction paragraph 5: Although uranium is weakly radioactive, its primary health risk stems from its chemical toxicity rather than its radioactivity. Chronic exposure to uranium-contaminated water is associated with nephrotoxicity [14 , 15], with adverse renal effects reported in both laboratory animals and humans [16]. Uranium excretion in urine is correlated with phosphate and calcium excretion [16]. Other adverse effects include inhibition of bone function and development [17], reproductive and developmental toxicity [18].

 3. Interpretation of Results

 a. The conclusion is too brief and lacks interpretation.

AUTHOR RESPONSE: We have expanded the conclusion section to discuss the scope, novelty, and utility of our data.

 b. The authors should describe the spatial distribution of uranium (e.g., “localized in the NW region”).

AUTHOR RESPONSE: We have provided a kernel density estimate of the groundwater uranium

 concentrations in and around Chintamani village (Figure 1B) that quantifies the spatial distribution of uranium.

 c. Discuss whether the observed concentrations exceed WHO or Indian drinking water standards.

AUTHOR RESPONSE: We have discussed this in the Conclusion section (paragraph 1):

 Conclusion paragraph 1: According to World Health Organization (WHO) recommendations [ 9] and Atomic Energy Regulatory board (AERB) recommendations, the uranium concentration in drinking water should remain ≤30 ppb (0.03 ppm)  and ≤60 ppb (0.06 ppm) respectively  to minimize health risks. 10 out of the 12 borewells sampled possessed uranium concentrations >0.03 ppm and 97 wells possessed uranium concentrations with >,0.06 ppm, indicating cause for concern. Uranium concentrations >0.03 ppm were observed both in the northwest region (borewells 1,6-12) as well as outside (borewells 4,5), indicating a wide distribution across the water table of Chintamani village. Borewell 8 possessed 8.64 ppm uranium, a concentration 288× greater than the WHO recommended maximum.

 d. Highlight any environmental or public health implications.

AUTHOR RESPONSE: Currently, this study does not report the environmental or public health effects of groundwater uranium in Chintamani village. We believe that further environmental / patient data from Chintamani village needs to be collected before commenting on these issues. We have mentioned this in the Conclusion (paragraph 4)

 Conclusion paragraph 4:  Further work is required to determine the environmental and public health effect of groundwater uranium in Chintamani village and its surroundings.

 4. Methodological Clarifications

 a.  The “Sample collection” section should include:

 i. GPS coordinates of all borewells (only partially included in Table 1).

AUTHOR RESPONSE: Latitudes and longitudes for all 12 samples are included in Table 1.

 ii. Clarification on how borewells were selected (random, purposive, based on prior data?).

AUTHOR RESPONSE: Borewells were initially randomly selected across the area in and around Chintamani village. Further samples were collected from the area around the borewell possessing the highest uranium concentration (Borewell 1). This has now been mentioned in the Introduction (paragraph 5) and Datasets section (paragraph 1).

 Introduction paragraph 5: “In this data note, we report data obtained from five randomly selected borewells in Chintamani village, Chikkaballapura District, Karnataka, India. Subsequently, seven additional borewells were selected in the vicinity of the borewell exhibiting the highest uranium concentration among the initial five. “

 Datasets  paragraph 1: Groundwater from 12 borewells in Chintamani village, Chikkaballapura District, Karnataka, India were sampled from August 2024 to December 2024. Initially, we collected groundwater samples from borewells 1-5 that were evenly distributed around the geographical area of Chintamani village. Groundwater from borewell 1 displayed the highest uranium con- centration from this cohort (0.771 ppm U), leading us to sample groundwater from more borewells around borewell 1 in the northwestern region of Chintamani village. 

 iii. The use of “t.test()” in the text should be corrected or explained more clearly.

AUTHOR RESPONSE: We have corrected **t.test()** to **t.test** and also provided a short explanation in the Methods subsection “Data representation and statistical analyses”.

 An explanation of the t.test function is provided in the same subsection, immediately before t.test is mentioned.

 “The **Welch 2-sample T-test (one-tailed) **was used to determine whether there existed a statistically significant difference between uranyl concentrations in different spatial locations in Chintamani village (Figure 1). This was performed using the function t.test. “

 5. Data Presentation

 a. The tables are comprehensive but dense.

 Author response provided under query 5d:

 b. Consider summarizing key findings in a visual format (e.g., heatmap, scatter plot).

 Author response provided under query 5d:

 c. Highlight borewells that exceed safety thresholds.

AUTHOR RESPONSE: 10 of the 12 borewells possessed uranium concentrations exceeding the World Health Organization (WHO) recommendation ≤0.03 ppm U). 9 of 12 borewells exceeded the atomic energy regulatory board (AERB) recommendation of (≤0.06 ppm U). We have highlighted these borewells in Table 1 (in bold and underlined font). To avoid visual clutter, we have only highlighted these wells in Table 1 and not in Figure 1A or 1B.  

 d. A spatial map showing uranium concentration gradients would enhance clarity.

AUTHOR RESPONSE for 5a, 5b, and 5d: We agree that the Tables 1 and 2 are very information dense. Our key findings are reported in Table 1 (groundwater uranium concentration for each borewell). We have now depicted this data in a visual format. We have added a local kernel density estimate (KDE) smoothing to predict area-wide uranium concentrations. This data is provided in Figure 1B.

 6. Language and Style

 Minor grammatical and typographical issues should be corrected:

 Example: “Forevery” → “For every”

AUTHOR RESPONSE: All instances of “forevery” have been changed to “for every” in the manuscript.

 Example: “hot-air ovenat” → “hot-air oven at”

AUTHOR RESPONSE: We have corrected this statement in the Methods subsection “Total dissolved solids (TDS) quantification”. It now reads:

 For every borewell sample, 1 L of water was dried in a hot-air oven at 80◦C in a clean Borosil ® borosilicate 1L glass beaker until only the salt residue remained. “

 Example: “t.test()” → should be formatted correctly or described in plain language.

AUTHOR RESPONSE: We have corrected **t.test()** to **t.test** and also provided a short explanation in the Methods subsection “Data representation and statistical analyses”. An explanation of the t.test function is provided in the same subsection, immediately before t.test is mentioned.

 “The **Welch 2-sample T-test (one-tailed) **was used to determine whether there existed a statistically significant difference between uranyl concentrations in different spatial locations in Chintamani village (Figure 1). This was performed using the function t.test. “

 Further Suggestions

 To enhance the manuscript’s scientific value and clarity:

 a. Expand the conclusion to include:

 b. A summary of spatial trends.

AUTHOR RESPONSE: The conclusion (paragraph 1) now contains a summary of spatial trends.

 c. Implications for public health or groundwater management.

 d. Recommendations for future research or monitoring.

AUTHOR RESPONSE to c/d: The conclusion (paragraph 4) now touches upon this issue. Further work is required before making public health recommendations.

 e. Add a brief section on limitations:

 f. Small sample size (12 borewells).

 g. Lack of temporal data (single sampling period).

 h. No direct health or exposure assessment.

AUTHOR RESPONSE to e/f/g/h: We have addressed these limitations in the conclusion, paragraphs 4 and 5:

 Conclusion (paragraph 4): Our study provides greater insights into groundwater uranium concentrations in Chintamani village, but is nevertheless limited by our larger but still modest sample size (n=12) and lack of temporal data.

 Conclusion (paragraph 5): Further work, such as direct health or exposure assessments, is required to determine the environmental and public health effect of groundwater uranium in Chintamani village and its surroundings.

 Include a visual summary (e.g., map or infographic) to improve accessibility.

AUTHOR RESPONSE: We have provided a visual summary in Figure 1B.

# Reviewer report: Reviewer response for version 1

**DOI:** 10.5256/f1000research.178738.r376222

**Published:** 2025-04-29

**Authors:** Wasiu Mathew Owonikoko

**Affiliations:** 1 Igbinedion University, Okada, Nigeria

The work titled “The distribution of groundwater uranium in Chintamani village, Karnataka, India” assessed the groundwater abundance of uranium in Chintamani village in India. The study is well-written with very minimal error and data well analyzed and interpreted. The comments of the reviewer, resolved along minor and major lines, are as stated below

Minor comment

- Under “Sample collection” in “Material and method” section, the coordinates/GPS location of the site should be provided
- “t.test()” in the second sentence of the first line in page 7 should be written correctly and the bracket filled or removed
- The conclusion should be beefed up to include information on the observed nature of the distribution of uranium in the village. i.e The distribution is of uranium in the village should be properly described. Is is widespread, low or high with the possible and attendant environmental and systemic implications, authors should state.
- Authors agreed that groundwater uranium concentration had been reported in India but did not impress why such investigation is being repeated within the same Country. Is the extant data now old, in a different area from the location of the last report or for merely confirmatory purpose?

Major comment

- The audience of this journal may consider the scope of the study limited. Authors, in the introduction section, agreed that the study is confirmatory; implying that the observation in this study is not new and has been earlier reported. As an advancement of previous report, it will be expected that authors add more information that answer vital questions such as:
- 1. What is/are the possible exposure pathways to ground water uranium among humans and animals.
- 2. What is the health risk associated with the exposure to uranium particularly via the gastrointestinal and dermal routes?
- 3. What is the systemic toxicological implication of the exposure to uranium

Are sufficient details of methods and materials provided to allow replication by others?

Partly

Is the rationale for creating the dataset(s) clearly described?

Yes

Are the datasets clearly presented in a useable and accessible format?

Yes

Are the protocols appropriate and is the work technically sound?

Yes

Reviewer Expertise:

Environmental Physiology and Toxicology

I confirm that I have read this submission and believe that I have an appropriate level of expertise to confirm that it is of an acceptable scientific standard, however I have significant reservations, as outlined above.

# Response

**Published:** 2025-05-19

**Authors:** Deepesh Nagarajan

The work titled “The distribution of groundwater uranium in Chintamani village, Karnataka, India” assessed the groundwater abundance of uranium in Chintamani village in India. The study is well-written with very minimal error and data well analyzed and interpreted. The comments of the reviewer, resolved along minor and major lines, are as stated below

**AUTHOR RESPONSE:** We thank the reviewer for his encouraging comments, and have addressed all his comments below.

Minor comment

- Under “Sample collection” in “Material and method” section, the coordinates/GPS location of the site should be provided
**AUTHOR RESPONSE:** We have provided the coordinates (latitude / longitude) for every sample in Table 1. We have now referred to Table in the sample collection section. “ Table 1 lists the latitude, longitude, and date of collection for every sample.”
- “t.test()” in the second sentence of the first line in page 7 should be written correctly and the bracket filled or removed
**AUTHOR RESPONSE: **We agree with the reviewer and have removed the () in t.test. The sentence now reads: “ This was performed using the function t.test”
- The conclusion should be beefed up to include information on the observed nature of the distribution of uranium in the village. i.e The distribution is of uranium in the village should be properly described. Is it widespread, low or high with the possible and attendant environmental and systemic implications, authors should state.
**AUTHOR RESPONSE: **We agree with the reviewer and have added the following material to the **conclusion**, discussing the distribution and systemic health effects of uranium:
Uranyl concentrations ranged from 0.018 ppm (borewell 2) to 8.64 ppm (borewell 8). According to World Health Organization (WHO) recommendations , uranium concentration in drinking water should remain ≤30 ppb (0.03 ppm)  to minimize health risks. 10 out of the 12 borewells sampled possessed uranium concentrations >0.03 ppm, indicating cause for concern. Uranium concentrations >0.03 ppm were observed both in the northwest region (borewells 1,6-12) as well as outside (borewells 4,5), indicating a wide distribution across the water table of Chintamani village. Borewell 8 possessed 8.64 ppm uranium, a concentration 288× greater than the WHO recommended maximum.
Nephrotoxicity , bone function impairments , developmental and reproductive toxicity are known adverse health effects associated with chronic uranium exposure. It is therefore worth studying the prevalence of such health effects in the residents of Chintamani village.
- Authors agreed that groundwater uranium concentration had been reported in India but did not impress why such investigation is being repeated within the same Country. Is the extant data now old, in a different area from the location of the last report or for merely confirmatory purposes?
**AUTHOR RESPONSE: **We thank the reviewer for raising an important point. R. Srinivasan et al. (reference 19) previously reported high uranium concentrations in groundwater in parts of eastern Karnataka (a state in India). Their study was broad in scope, reporting uranium concentrations from 73 separate villages in the state. Due to the broad scope, only 1-2 samples per village were collected. The authors did not report the exact location of sample collection for every village.
  Our study is narrower in scope, as we survey the spatial distribution of groundwater uranium concentration in a single village (Chintamani). We show that uranium concentrations in groundwater can greatly vary even within the water table of a single village. R. Srinivasan et al. reported uranium concentrations of 5267 ± 6 ug/g and  5913 ± 6 uranium from 2 borewells sampled in Chintamani. Here, we show a far greater variation in uranium concentrations, ranging from 0.018 ppm to 8.64 ppm (mean = 1.39 ±2.55 ppm), from the 12 borewells we sampled in Chintamani.

Our two studies therefore have different scopes and complement each other. This has now been mentioned in the **Conclusion** section.

Major comment

- The audience of this journal may consider the scope of the study limited. Authors, in the introduction section, agreed that the study is confirmatory; implying that the observation in this study is not new and has been earlier reported. As an advancement of previous report, it will be expected that authors add more information that answer vital questions such as:
- 1. What is/are the possible exposure pathways to ground water uranium among humans and animals.
**AUTHOR RESPONSE:** We thank the reviewer for raising an important point. As Chintamani is an agricultural village, we believe it’s pertinent to discuss uranium uptake in plants in addition to animals and humans. We have now discussed these issues in the **introduction **section.
- 2. What is the health risk associated with the exposure to uranium particularly via the gastrointestinal and dermal routes **?**
** AUTHOR RESPONSE:** Dermal exposure results in minimal toxicity, particularly if Uranium is in an insoluble form. Uranium in groundwater is typically present as sparingly soluble uranyl carbonate, minimizing the risk of absorption through the dermal route.
  Oral consumption of uranium leads to nephrotoxicity, inhibition of bone function and development, reproductive and developmental toxicity. These points are now discussed in the **introduction **section.
- 3. What is the systemic toxicological implication of the exposure to uranium?
**AUTHOR RESPONSE:**  Oral consumption of uranium-contaminated groundwater leads to systemic toxicological effects: nephrotoxicity, inhibition of bone function and development, reproductive and developmental toxicity. These points are discussed in the **introduction** section alongside health risks associated with oral consumption (previous comment).

**AUTHOR RESPONSE:**  The relevant excerpt for the introduction section covering **Major comment points 1-3** is provided below:

Uranium adversely affects crops grown on soil irrigated with contaminated groundwater. Uranium’s phytotoxic effects include inhibition of photosynthesis, inhibition of plant growth, protein and lipid membrane oxidation, overproduction of reactive oxygen species, and DNA breakage. Uranium primarily accumulates in root systems, with negligible amounts found in aerial parts of plants and is therefore a greater concern for root and tuber crops.

Dermal exposure results in minimal toxicity, particularly if Uranium is in an insoluble form. Uranium in groundwater is typically present as sparingly soluble uranyl carbonate complexes, minimizing the risk of absorption through the dermal route. Insteal, oral consumption of uranium through drinking untreated uranium-contaminated groundwater is of more concern. The World Health Organization (WHO) recommends a maximum uranium concentration of 30 ppb in drinking water to minimize health risks. Although uranium is weakly radioactive, its primary health risk stems from its chemical toxicity rather than its radioactivity. Chronic exposure to uranium-contaminated water is associated with nephrotoxicity , with adverse renal effects reported in both laboratory animals and humans. Uranium excretion in urine is correlated with phosphate and calcium excretion. Other adverse effects include inhibition of bone function and development, reproductive and developmental toxicity.

- Is the rationale for creating the dataset(s) clearly described?
  Yes
- Are the protocols appropriate and is the work technically sound?
  Yes
- Are sufficient details of methods and materials provided to allow replication by others?
  Partly
- Are the datasets clearly presented in a useable and accessible format?
  Yes

**Competing Interests**

 No competing interests were disclosed.

**Reviewer Expertise**

 Environmental Physiology and Toxicology

**I confirm that I have read this submission and believe that I have an appropriate level of expertise to confirm that it is of an acceptable scientific standard, however I have significant reservations, as outlined above.**

**AUTHOR RESPONSE: **We thank the reviewer for his meticulous feedback, and we hope to have addressed all the concerns raised in this response.
